# Low phospholipid associated cholelithiasis: association with mutation in the *MDR3*/*ABCB4 *gene

**DOI:** 10.1186/1750-1172-2-29

**Published:** 2007-06-11

**Authors:** Olivier Rosmorduc, Raoul Poupon

**Affiliations:** 1 Service d'Hépatologie, INSERM U 680, Centre de Référence de Maladies Rares et des Maladies Inflammatoires des Voies Biliaires; Hôpital Saint-Antoine, Assistance Publique-Hôpitaux de Paris; Faculté de Médecine Pierre et Marie Curie et Université Paris 6; Paris, France

## Abstract

Low phospholipid-associated cholelithiasis (LPAC) is characterized by the association of *ABCB4 *mutations and low biliary phospholipid concentration with symptomatic and recurring cholelithiasis. This syndrome is infrequent and corresponds to a peculiar small subgroup of patients with symptomatic gallstone disease. The patients with the LPAC syndrome present typically with the following main features: age less than 40 years at onset of symptoms, recurrence of biliary symptoms after cholecystectomy, intrahepatic hyperechoic foci or sludge or microlithiasis along the biliary tree. Defect in ABCB4 function causes the production of bile with low phospholipid content, increased lithogenicity and high detergent properties leading to bile duct luminal membrane injuries and resulting in cholestasis with increased serum gamma-glutamyltransferase (GGT) activity. Intrahepatic gallstones may be evidenced by ultrasonography (US), computing tomography (CT) abdominal scan or magnetic resonance cholangiopancreatography, intrahepatic hyperechogenic foci along the biliary tree may be evidenced by US, and hepatic bile composition (phospholipids) may be determined by duodenoscopy. In all cases where the *ABCB4 *genotyping confirms the diagnosis of LPAC syndrome in young adults, long-term curative or prophylactic therapy with ursodeoxycholic acid (UDCA) should be initiated early to prevent the occurrence or recurrence of the syndrome and its complications. Cholecystectomy is indicated in the case of symptomatic gallstones. Biliary drainage or partial hepatectomy may be indicated in the case of symptomatic intrahepatic bile duct dilatations filled with gallstones. Patients with end-stage liver disease may be candidates for liver transplantation.

## Disease name and synonyms

Low phospholipid associated cholelithiasis

Cholelithiasis with *ABCB4 *gene mutation

*ABCB4 *gene mutation-associated cholelithiasis

## Definition

Low phospholipid-associated cholelithiasis (LPAC) is characterized by the association of *ABCB4 *mutations with symptomatic and recurring cholelithiasis in young adults (*e.g. *<40 years).

## Epidemiology

The exact prevalence of LPAC remains unknown. The disease is more common in young adults, the usual age at the onset of the symptoms is typically lower than 40 years. The male to female ratio is estimated at approximately 1:3. However, at present, the literature data and our results did not allow to determine the frequency of *ABCB4 *mutations in the whole population of patients with cholelithiasis.

Up to 10% of the European and American population carry gallstones, approximately 25% of cases have symptoms and less than 2% present with severe complications (cholangitis or pancreatitis). Based on previous epidemiological data, we consider that LPAC syndrome is infrequent and corresponds to a peculiar subgroup of patients with symptomatic gallstone disease [1,2]. Considering the advances in molecular diagnosis of the disease, the undiagnosed cases could be identified and provide data about the prevalence of LPAC.

## Clinical description

LPAC presents as a peculiar form of cholelithiasis characterized by cholecystitis, cholangitis and intrahepatic gallstone disease, and/or acute pancreatitis associated with biliary microlithiasis. Most patients report a history of cholesterol gallstones amongst their first-degree relatives. Intrahepatic hyperechoic foci are characteristic sign of the LPAC syndrome. Intrahepatic gallstones or sludge and increased serum gamma-glutamyl transferase (GGT) activity are frequently present. It suggests that the biliary symptoms experienced by these patients are probably caused by cholesterol crystal deposits and bile duct inflammation but not directly and exclusively related to the presence of detectable gallstones. Increased cholesterol saturation index and a defect in the hepatic transport and biliary secretion of phospholipids in patients with an intrahepatic cholesterol gallstone disease or an acute pancreatitis associated with biliary microlithiasis have been shown in another studies [3,4].

The typical biliary pain in patients with LPAC syndrome (associated with detectable gallstones or not) often leads to decision of cholecystectomy. The majority of patients is our series (90%) underwent cholecystectomy but recurrence of the symptoms was observed in half of them, despite cholecystectomy [5]. The onset of symptoms typical for LPAC syndrome have been reported at the end of or following pregnancy (56% of women with early onset of symptoms and *ABCB4 *mutations presented also with an history of intrahepatic cholestasis of pregnancy and 14% had fetal complications) [6]. Prophylactic therapeutic activity of ursodeoxycholic acid (UDCA) has been demonstrated in the majority of patients [5]. Of note, the benefit of UDCA therapy on the resolution of biliary symptoms occurred long before the dissolution of the intrahepatic stones. Therefore, recurrence despite cholecystectomy, association with intrahepatic cholestasis of pregnancy and prevention by UDCA represent major clinical features that support the diagnosis of LPAC syndrome.

## Etiology

Phospholipids are the major carrier and solvent of cholesterol and they exert a protective effect against bile salt-induced biliary mucosa injury. Homozygous and heterozygous mutations in the *MDR3 *(now referred to as *ABCB4*) gene, the phosphatidylcholine translocator across the canalicular membrane, are thought to be responsible for progressive intrahepatic cholestasis type 3 (PFIC 3) [7], and heterozygous mutations have been also reported in patients presenting with a history of intrahepatic cholestasis of pregnancy or with a cholangiopathy referred to as anti-mitochondrial antibody (AMA) negative primary biliary cirrhosis (PBC) [8-11]. Defect in ABCB4 function causes the production of bile with low phospholipid content, increased lithogenicity and high detergent properties leading to bile duct luminal membrane injuries and resulting in cholestasis with increased serum GGT activity.

**Figure 1:**
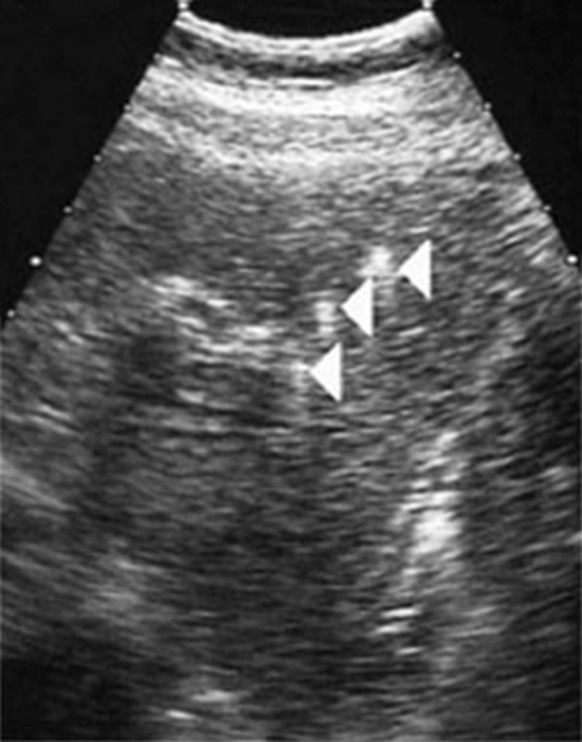
Ultrasound images of intrahepatic hyperechoic foci in patients with the LPAC syndrome. In these patients, a careful US examination detected intrahepatic hyperechoic foci with diffuse topography compatible with lipid deposits along the luminal surface of the intrahepatic biliary tree (Arrows). These multiple dots, less than 1 mm in diameter, cast short echogenic trail without acoustic shadows and looked like comet tails. They were typically distributed along the portal arborizations and may be associated with intrahepatic sludge or microlithiasis casting typical acoustic shadows.

We determined more generally the frequency of *ABCB4 *gene mutations in 60 consecutive adult patients who had been referred to our liver unit because of symptomatic or complicated cholelithiasis and we characterized more precisely the clinical phenotype associated with these mutations [12]. Among these patients, 32 were referred specifically for *ABCB4 *gene analysis because of clinical history compatible with the syndrome that we previously described (*i.e. *presenting at least 3 of the following clinical criteria: age at the onset of symptoms lower than 40 years; association with cholecystitis, cholangitis or acute pancreatitis; recurrence of symptoms despite cholecystectomy; intrahepatic hyperechoic foci (Figure 1) with or without intrahepatic sludge or microlithiasis). Twenty-eight of these patients were referred after a cholecystectomy for a symptomatic gallstone disease and 33 other consecutive patients were referred for diverse acute or chronic liver diseases without biliary gallstones (chronic HBV or HCV hepatitis, non-alcoholic steatohepatitis, drug-induced hepatitis or a classic genetic hemochromatosis).

### Clinical phenotype associated with *ABCB4 *gene mutations

Among the 32 patients suspected of having the syndrome, 18 (56%, CI 95% [52%–62%]) presented a point mutation at the *ABCB4 *locus, while none of the 28 patients with a classical form of cholelithiasis and none of the 33 patients without cholelithiasis had mutation in the *ABCB4 *gene (p < 0,001 and p < 0,0001; respectively). Multivariate analysis showed that among patients with cholelithiasis three independent factors were predictive of a mutation at the *ABCB4 *locus: a recurrence of symptoms after cholecystectomy (adjusted OR = 8.5), intrahepatic hyperchoic material (adjusted OR = 6.1), and age <40 years (adjusted OR = 3.0) [9].

Finally, more recent data shows that the LPAC syndrome is more frequent in females and that biliary symptoms occur earlier in female who present a nonsense mutation [6].

### Characterization of the *ABCB4 *gene mutations in patients with the LPAC syndrome

**Figure 2:**
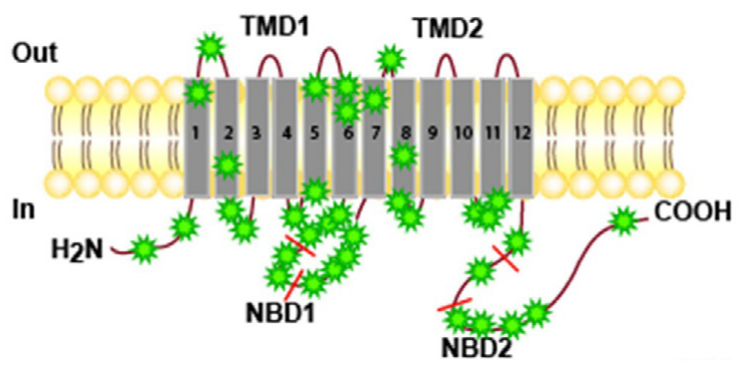
Localization of the DCMs in the different domains of the ABCB4 protein in the complete series of patients with LPAC.

Patients were screened for mutations in the *ABCB4 *gene using polymerase chain reaction (PCR) amplification and DNA sequencing of exons 2 to 28 and all splice junctions. We identified 14 heterozygous and homozygous point mutations amongst these 18 patients. None of these mutations was detected in a control panel of 140 chromosomes demonstrating that they did not correspond to polymorphisms (SNPs). Affected patients with heterozygous mutations exhibited 1bp-insertion, 1bp-deletion, nonsense mutations or missense mutations resulting in a frameshift predicted to cause premature messenger RNA termination, a loss of protein function and single-nucleotide substitutions, while affected patients with homozygous mutations demonstrated only missense mutations. Most mutations were localized in the central part of the molecule, close to nucleotide binding domain 1 (NBD1), or in adjacent transmembrane domains and intracellular loops (Figure 2). Eighty percent of mutations were indeed situated in regions encoded by exons 9 to 18, which corresponded approximately to 38% of the encoding region (TM 5 and 6; 3^rd ^intracellular loop including NBD1; TM 7 and 8).

### Possible disease-causing mutation

The definition of a disease-causing mutation (DCM) is problematic when functional assays to determine the phenotypic effects of specific variants are not available or have not been performed. To date, two of the missense mutations (Glu528Asp and Thr175Ala) detected in patients with the LPAC syndrome had been analyzed previously in a homolog of ABCB4 (Pgp now called ABCB1) in yeast [13,14]. The Thr175Ala mutation was localized in the 1^st ^intracellular loop and resulted in a substitution in a conserved cluster of four amino-acids at position 169–172, required for the adenosine triphosphatase activity of the molecule. This mutant resulted in a complete loss of drug-induced P-gp ATPase activity. The Phe165Ile mutation was localized in the same part of this intracellular loop and may therefore give rise to a similar defect. The Glu528Asp mutation was localized close to the NBD1. The glutamic acid is included in a small amino-acid cluster localized before the walker B motif which is required for drug-induced ATPase activity. These mutations indeed induced a loss of function of the molecule *in vitro*. It should be noted that the two unrelated patients in whom the Thr175Ala mutation was identified originated from Northern Europe, so they may have inherited a founder mutation from a shared ancestor. This mutation has also been recently identified in adults with cholangiopathy and intrahepatic cholestasis of pregnancy [11]. The mutation S320F has also been recently identified in a patient with intrahepatic cholestasis of pregnancy and cholelithiasis [7,11].

Only *ABCB4 *sequence alterations leading to predicted premature truncation of the protein, small deletions or insertions and non-conserved missense mutations were considered as potential DCMs. Even if this restriction was taken into account, a certain number of arguments strongly supported a pathogenic role for *ABCB4 *gene mutations. These mutations are indeed detected at high frequency in patients with LPAC syndrome; they affect only highly conserved amino acids between human and rodent homologues of the gene and no mutation was detected in an independent control panel of 140 chromosomes. In addition, LPAC syndrome was observed in patients exhibiting the same or similar nonsense or missense mutations: in a mother and her elder son with an identical heterozygous nonsense mutation (1327insA); in independent patients from non-consanguineous families with the same homozygous missense mutation (Ser320Phe or Ala934Thr), while their heterozygous parents were asymptomatic; in patients with a similar nonsense mutation (1006-1016insT and 1006-1016delT) and in unrelated patients with the same missense mutations (Pro1161Ser). For all these reasons, these mutations could be considered as causing the LPAC syndrome.

These results and data in the literature therefore strongly suggest that *ABCB4 *mutations may lead to cholesterol cholelithiasis when residual *ABCB4 *activity and subsequent biliary phospholipid secretion fall below a critical threshold which may depend on the type of mutation and on other host or environmental factors, including probably bacterial infection of the bile ducts (Carey *et al*. Personnal Communication. Falk Symposium 2004).

### Alternative molecular mechanisms of LPAC syndrome

The mutation screening method used in our two studies was unable to detect major DNA rearrangements, and nor did the analysis include the promoter or other potential regulation regions of the gene. As 44% of LPAC patients did not present *ABCB4 *gene point mutation, defects in the promoter region or large DNA rearrangements might also be involved in some patients presenting with LPAC syndrome. Alternatively, defects in other regions of the gene or in other genes may also be involved, and some evidence from animal studies has pointed to *Abcb *11 (previously called the bile salt export pump, *BSEP*), *Abcc *2 (previously referred to as multidrug resistance related protein 2, or *MRP2*) or *Abcg5*/*Abcg8 *as other possible candidate genes underlying susceptibility to cholelithiasis [15-19] or phospholipid secretion disruption [20].

### Animal models of the LPAC syndrome

It has also been shown that substantial amounts of cholesterol are secreted by Mdr2 (-/-) mice on a cholate-supplemented diet and microscopic examination of the gallbladder reveals massive amount of cholesterol crystals and that after 12 weeks, 50% of the Mdr2 (-/-) mice on chow also developed gallstones composed of needlike "anhydrous" cholesterol crystals and mucin [21,22]. Indeed, although bile of these mice contain traces only of cholesterol, they are supersaturated with cholesterol (CSI ≥ 1.05) and intrahepatic bile duct are filled with crystalline occlusions composed of needle-like crystals plus mucin gel in 7-month-old mice. Female Mdr2 -/- mice display a more hydrophobic bile salt pool due to a lower tauro-beta-muricholate level in bile and increasing bile hydrophobicity has been demonstrated *in vitro *to enhance cholesterol crystallization. This mechanism explains the early and higher prevalence of gallstones in female Mdr2 -/- mice and provide a rationale for substitution of more hydrophobic bile salts with ursodeoxycholic acid in LPAC patients [21].

### *ABCB4 *gene mutations and primary sclerosing cholangitis

As a recent study reported that Mdr2 KO mice develop liver lesions mimicking sclerosing cholangitis characterized by biliary strictures and dilatations [23], we also looked for *ABCB4 *gene mutations and sequence diversity in 34 consecutive patients with primary sclerosing cholangitis. We found no *ABCB4 *gene mutations and no abnormal SNP frequency in these unselected PSC patients [24]. These data are consistent with the results of the recent study of the group from Zurich which observed no significant deviation in haplotype structure in *ABCB4 *from that observed in healthy Caucasian controls [25]. Thus, these results, unlike the bile duct lesions observed in mice, do not support a major role of *ABCB4 *genetic variation in the pathogenesis of the PSC, although an implication of *ABCB4 *cannot be ruled out in a subgroup of these patients.

### Cholelithiasis and *ABCB4 *gene mutation-associated liver diseases in adults

**Figure 3:**
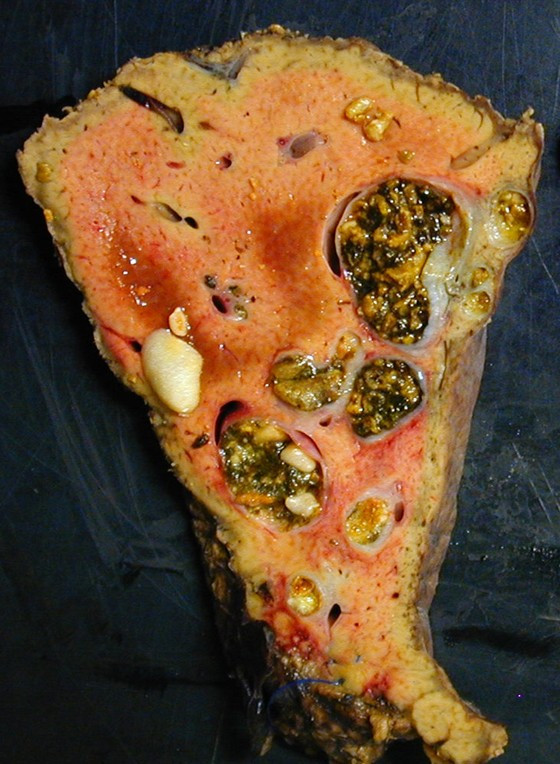
LPAC syndrome associated with the presence of intrahepatic non cystic bile duct dilatations filled with cholesterol gallstones.

Cholesterol cholelithiasis was also demonstrated in patients with progressive familial intrahepatic cholestasis-3 (PFIC3) and some of their relatives [7,26], and in patients with a clinical presentation of intrahepatic cholestasis of pregnancy [27-30]. In addition, a multidrug resistance 3 gene mutation in exon 14 has been recently detected in a patient who developed cholelithiasis in adolescence, followed by cholestasis of pregnancy and finally adulthood biliary cirrhosis [31]. Finally, we also observed in some patients presenting with the LPAC syndrome the presence of localized or diffuse intrahepatic non cystic bile duct dilatations filled with cholesterol gallstones (Figure 3). These peculiar bile duct dilatations as well as some cases of adulthood biliary cirrhosis may therefore be considered as severe complications of the LPAC syndrome.

## Diagnosis and diagnostic methods

In patients with symptomatic cholelithiasis, the presence of additional hepatobiliary manifestations (age at onset of biliary symptoms, intermittent cholestatic attachs, material or sludge in the intrahepatic bile ducts, recurrence of biliary symptoms after cholecystectomy *etc.*) should remind of this rare monogenic form of cholelithiasis (*e.g. *LPAC syndrome) for which genetic testing may be offered by several genetic laboratories.

### Diagnostic criteria

The LPAC syndrome should be supposed in cases with clinical history of symptomatic cholelithiasis with at least one of the following criteria: age less than 40 years at onset of symptoms, recurrence after cholecystectomy, intrahepatic hyperechoic foci with a topography compatible with lipid deposits along the luminal surface of the intrahepatic biliary tree, intrahepatic sludge, microlithiasis, familial history of cholelithiasis in first-degree relatives, or clinical history of intrahepatic intrahepatic cholestasis of pregnancy.

### Diagnostic methods

• Intrahepatic gallstones evidenced by ultrasonography (US), computing tomography (CT) abdominal scan or magnetic resonance cholangiopancreatography;

• Intrahepatic hyperechogenic foci along the biliary tree by US;

• Hepatic bile composition (obtained by duodenoscopy and aspiration using a catheter introduced in the distal common bile duct or by T-tube after surgery);

• *ABCB4 *gene analysis.

### Differential diagnosis

Other inflammatory bile duct diseases which may lead to biliary symptoms and/or intrahepatic gallstones (including Caroli disease, primary scherosing cholangitis).

## Genetic counseling

*ABCB4 *genotyping should be used to confirm the diagnosis of LPAC syndrome in young adults who present with a symptomatic cholelithiasis and should allow familial screening.

## Management

### Non-surgical treatment

Special diet is not required. In all cases where the *ABCB4 *genotyping confirms the diagnosis of LPAC syndrome in young adults, long-term curative or prophylactic therapy with UDCA should be initiated early to prevent the occurrence or recurrence of the syndrome and its complications. UDCA was shown to up-regulate the expression of the protein at the canalicular membrane, minimize the toxicity of the endogenous hydrophobic bile acids and increase the pool of protective hydrophilic bile acids.

In the case of associated hypercholesterolemia, statins should be preferred to fibrates which increase the lithogenicity of the bile.

### Surgical treatment

Cholecystectomy is indicated in the case of symptomatic gallstones but not when only sludge is present in the gallbladder.

Biliary drainage or partial hepatectomy may be indicated in the case of symptomatic intrahepatic bile duct dilatation filled with gallstones.

Patients with end-stage liver disease may be candidates for liver transplantation.

### Future pharmacological treatment

Drugs that may induce ABCB4 expression.

## Clinical course and outcome

Typically, the clinical course is progressive, with multiple recurrence despite the operative intervention(s) until UDCA treatment is started.

The majority of patients with LPAC syndrome described in the literature do not develop recurrence or end-stage liver disease under medical treatment.

## Conclusion

In summary, our results strongly support the role of an *ABCB4 *gene defect in LPAC syndrome and replace it in the context of *ABCB4 *gene-associated liver diseases in adults including attenuated form of PFIC 3, "atypical intrahepatic cholestasis of pregnancy", LPAC syndrome which might lead to adulthood biliary cirrhosis or bile duct dilatations associated with gallstones. A genetic test based on the present results may permit the molecular diagnosis of LPAC syndrome and the screening of high-risk subjects. Depending on the results, long-term curative or prophylactic UDCA therapy may be initiated early to prevent the occurrence or recurrence of this syndrome and its severe complications.

## References

[B1] Diehl AK (1992). Symptoms of gallstone disease. Baillieres Clin Gastroenterol.

[B2] Diehl AK, Schwesinger W, Holleman DJ, Chapman J, Kurtin W (1995). Clinical correlates of gallstone composition : distinguishing pigment from cholesterol stones. Am J Gastroenterol.

[B3] Fracchia M, Pellegrino S, Secreto P, Gallo L, Masoero G, Pera A, Galatola G (2001). Biliary lipid composition in cholesterol microlithiasis. Gut.

[B4] Shoda J, Oda K, Suzuki H, Sugiyama Y, Ito K, Cohen D, Feng L, Kamiya J, Nimura Y, Kano M, Matsuzaki Y, Tanaka N (2001). Etiologic significance of defects in cholesterol, phospholipid, and bile acid metabolism in the liver of patients with intrahepatic calculi. Hepatology.

[B5] Rosmorduc O, Hermelin B, Poupon R (2001). MDR3 gene defect in adults with symptomatic intrahepatic and gallbladder cholesterol cholelithiasis. Gastroenterology.

[B6] Rosmorduc O, Kedzia C, Boelle PY, Chazouillères O, Hermelin B, Poupon R (2005). Intrahepatic cholesterol cholelithiasis associated with ABCB4 gene mutations: phenotype-genotype relationship [abstract]. Hepatology.

[B7] Jacquemin E, De Vree M, Cresteil D, Sokal E, Sturm E, Dumont M, Scheffer G, Paul M, Burdelski M, Bosma P, Bernard O, Hadchouel M, Oude Elferink R (2001). The wide spectrum of multidrug resistance 3 deficiency: from neonatal cholestasis to cirrhosis of adulhood. Gastroenterology.

[B8] Dixon P, Weerasekera N, Linton K, Donaldson O, Chambers J, Egginton E, Weaver J, Nelson-Piercy C, Swiet M, Warnes G, Elias E, Higgins C, Johnston D, McCarthy M, Williamson C (2000). Heterozygous MDR3 missense mutation associated with intrahepatic cholestasis of pregnancy: evidence for a defect in protein trafficking. Hum Mol Genet.

[B9] Gendrot C, Bacq Y, Brechot M-C, Lansac J, Andres C (2003). A second heterozygous MDR3 nonsense mutation associated with intrahepatic cholestasis of pregnancy. J Med Genet.

[B10] Jacquemin E, Cresteil D, Manouvrier S, Boute O, Hadchouel M (1999). Heterozygous non-sense mutation of the MDR3 gene in familial intrahepatic cholestasis of pregnancy [letter]. Lancet.

[B11] Strautnieks S, Lopes A, Underhill J, Gerred S, Nibbering K, Knisely A, Portman B, Bomford A, Heaton N, Mieli-Vergani G, Oude Elferink R, O'Grady J, Wali S, Thompson R (2002). Critical residues in the multidrug resistance 3 protein gene associated with adult onset of cholangiopathy and intrahepatic cholestasis of pregnancy. Hepatology.

[B12] Rosmorduc O, Hermelin B, Boelle P, Parc R, Taboury J, Poupon R (2003). ABCB4 gene mutation-associated cholelithiasis in adults. Gastroenterology.

[B13] Hanna M, Brault M, Kwan T, Kast C, Gros P (1996). Mutagenesis of transmembrane domain 11 of P-glycoprotein by alanine scanning. Biochemistry.

[B14] Kwan T, Gros P (1998). Mutational analysis of the P-glycoprotein first intracellular loop and flanking transmembrane domains. Biochemistry.

[B15] Bouchard G, Nelson HM, Lammert F, Rowe LB, Carey MC, Paigen B (1999). High-resolution maps of the murine Chromosome 2 region containing the cholesterol gallstone locus, Lith1. Mamm Genome.

[B16] Bouchard G, Carey M, Paigen B (2000). In the genetic locus containing the second cholesterol gallstone gene Lith2, an Mrp2 polymorphism is linked to heightened secretion of organic anions [abstract]. Gastroenterology.

[B17] Khanuja B, Cheah YC, Hunt M, Nishina PM, Wang DQ, Chen HW, Billheimer JT, Carey MC, Paigen B (1995). Lith1, a major gene affecting cholesterol gallstone formation among inbred strains of mice. Proc Natl Acad Sci USA.

[B18] Lammert F, Carey M, Paigen B (2001). Chromosomal organisation of candidate genes involved in cholesterol gallstone formation: a murine gallstone map. Gastroenterology.

[B19] Wittenburg H, Lyons M, Renhua L, Churchill G, Carey M, Paigen B (2003). FXR and ABCG5/ABCG8 as determinants of cholesterol gallstone formation from quantitative trait locus mapping in mice. Gastroenterology.

[B20] Fouassier L, Kinnman N, Lefèvre G, Lasnier E, Rey C, Poupon R, Oude Elferink R, Housset C (2002). Contribution of mrp2 in alteration of canalicular bile formation by the endothelin antagonist bosentan. J Hepatol.

[B21] Lammert F, Wang DQ, Hillebrandt S, Geier A, Fickert P, Trauner M, Matern S, Paigen B, Carey MC (2004). Spontaneous cholecysto- and hepatolithiasis in Mdr2 -/- mice: a model for low phospholipid-associated cholelithiasis. Hepatology.

[B22] Oude Elferink R, Ottenhof R, van Wiijland M, Frijters C, van Nieuwkerk C, Groen A (1996). Uncoupling of biliary phospholipid and cholesterol secretion in mice with reduced expression of mdr2 P-glycoprotein. J Lipid Res.

[B23] Fickert P, Zollner G, Fuchsbichler A, Stumptner C, Weiglein A, Lammert F, Marschall H-U, Tsybrovsky O, Zatloukal K, Denk H, Trauner M (2002). Ursodeoxycholic acid aggravates bile infarcts in bile duct-ligated and Mdr2 knockout mice via disruption of cholangioles. Gactroenterology.

[B24] Rosmorduc O, Hermelin B, Boelle P, Poupon R, Poupon R, Chazouillères O (2004). ABCB4 gene mutations and primary sclerosing cholangitis. Gastroenterology.

[B25] Pauli-Magnus C, Kerb R, Fattinger K, Lang T, Anwald B, Kullak-Ublick G, Beuers U, Meier P (2004). Bsep and MDR3 haplotype structure in healthy caucasians, primary biliary cirrhosis and primary sclerosing cholangitis. Hepatology.

[B26] Jacquemin E (2001). Role of multidrug resistance 3 deficiency in pediatric and adult liver disease: one gene for three diseases. Seminars Liver Dis.

[B27] Simon E, Marschall H-U, Glantz A, Rath W, Matern S, Lammert F (2002). Mutations of hepatocanalicular ABC transporters in intrahepatic cholestasis of pregnancy. Hepatology.

[B28] Lammert F, Marschall H-U, Glantz A, Matern S (2000). Intrahepatic cholestasis of pregnancy: molecular pathogenesis, diagnosis and management. J Hepatol.

[B29] Van Dyke R, Zakim M, Boyer T (1996). The liver in pregnancy. Hepatology: A Textbook of Liver Disease 2.

[B30] Wasmuth HE, Glantz A, Keppeler H, Simon E, Bartz C, Rath W, Mattsson LA, Marschall HU, Lammert F (2006). Intrahepatic cholestasis associated with common variants of the hepatobiliary phospholipid transporter ABCB4 gene. Gut.

[B31] Lucena J-F, Herrero J, Quiroga J, Sangro B, Garcia-Foncillas J, Zabalegui N, Sola J, Herraiz M, Medina J, Prieto J (2003). A multidrug resistance 3 gene mutation causing cholelithiasis, cholestasis of pregnancy, and adulthood biliary cirrhosis. Gastroenterology.

